# Technical Proposal for Monitoring Thermal and Mechanical Stresses of a Runway Pavement

**DOI:** 10.3390/s21206797

**Published:** 2021-10-13

**Authors:** Salvatore Bruno, Giulia Del Serrone, Paola Di Mascio, Giuseppe Loprencipe, Eugenio Ricci, Laura Moretti

**Affiliations:** 1 Department of Civil, Constructional and Environmental Engineering, Sapienza University, Via Eudossiana, 18-00184 Rome, Italy; salvatore.bruno@uniroma1.it (S.B.); giulia.delserrone@uniroma1.it (G.D.S.); paola.dimascio@uniroma1.it (P.D.M.); giuseppe.loprencipe@uniroma1.it (G.L.); 2 DIAMONDS S.r.l., Via Raffaele Cadorna, 29-00187 Rome, Italy; eugenio.ricci@e-diamonds.it

**Keywords:** airport pavement, pavement management system, sensors, thermal stresses, mechanical stresses, monitoring system, remote system

## Abstract

Airport pavements should ensure regular and safe movements during their service life; the management body has to monitor the functional and structural characteristics, and schedule maintenance work, balancing the often conflicting goals of safety, economic and technical issues. This paper presents a remote monitoring system to evaluate the structural performance of a runway composed of concrete thresholds and a flexible central runway. Thermometers, strain gauges, and pressure cells will be embedded at different depths to continuously monitor the pavement’s response to traffic and environmental loads. An innovative system allows data acquisition and processing with specific calculation models, in order to inform the infrastructure manager, in real time, about the actual conditions of the pavement. In this way, the authors aim to develop a system that provides useful information for the correct implementation of an airport pavement management system (APMS) based on real-life data. Indeed, it permits comprehensive monitoring functions to be performed, based on the embedded sensing network.

## 1. Introduction

Pavements contribute to ensuring the safe and efficient operation of transport infrastructures [1,2]; they should be monitored over time, in order to prevent severe distresses due to the vertical and tangential forces transmitted by the aircraft, and thermo-hygrometric conditions [3]. The evaluation and prediction of the functional and structural performance of the pavements play a pivotal role in guaranteeing safety and regularity of operations [4]. The monitoring of runway evenness and adherence is necessary to analyze the structural response of the pavement to the applied loads, and predict its answer during its service life [5]. Direct measurements, visual investigations, or a combination of both allow the synthetic quality indices to be calculated. With regard to the surface characteristics of the pavement, the international roughness index (IRI), pavement condition index (PCI), British pendulum number (BPN), and Boeing bumping index (BBI) [6,7,8] are the most frequent indices. With regard to the bearing capacity, the aircraft classification number/pavement classification number (ACN/PCN) method [9], defined by the International Civil Aviation Organization (ICAO) [10], is adopted to quantify the maximum load that a pavement can bear (PCN) and the effect produced by an aircraft on a given pavement (ACN) [11]. Although the structural and functional performances of a pavement are related to each other, to date, no well-defined correlation is known [12], and these two pavement characteristics are separately assessed. Therefore, the implementation of an airport pavement management system (APMS) requires structural and functional approaches from infrastructure managers to maintain the pavement in a serviceable condition over a given period of time [13,14], and with the primary objective of reducing the risk of accidents [15,16,17,18]. A delay or failure to carry out maintenance work can increase the management costs, by raising the pavement deterioration rate, and impair the serviceability, by increasing aircraft accidents or delays in flight operations [19]. On the other hand, APMS is mandatory to obtain the airport certificate for the most trafficked aerodromes, according to [13].

The implementation of a pavement management system is necessary not only in the airport environment, but also in the heliport one, as shown by recent studies [20].

The most commonly used airport pavements are rigid and flexible pavements, which differ from each other in the transmission of forces to the subgrade in the following ways:

- Flexible pavement is composed of upper asphalt layers: it produces a uniform transmission of vertical stresses, which corresponds to a non-uniform distribution of vertical deformations;
- Rigid pavement is composed of concrete slabs: it produces a non-uniform transmission of vertical stresses, which corresponds to a uniform distribution of vertical deformations.

Whatever the type is, the pavement design and management should consider cyclic repetitions of traffic and thermal-hygrometric loads during the service life, in order to prevent structural failures [21]. For flexible pavements, the main distresses are as follows [22]: fatigue cracking of bonded layers, rutting of all pavement layers, and thermal cracking of surface layers. For rigid pavements, they are as follows [23]: concrete slab distress, joint faulting, and pumping. Periodic monitoring of the structural characteristics allows the identification of distress curves, to define deterioration prediction models of the pavement performance. The Benkelman beam [24,25] and falling weight deflectometer (FWD) [26] are traditional methods to evaluate the mechanical performance of a pavement; they closely reproduce stress–strain conditions and they show similar results in terms of deflection measurements [27]. It has to be noted that the surface deflections and moduli of the pavements are significantly influenced by temperature [28], and they need to be corrected. In particular, the results of these kind of surveys need to be back-calculated through deterministic or probabilistic approaches [29,30], to evaluate the performance of the pavements [31], in order to plan maintenance strategies for both roads and airport infrastructures [32].

However, these techniques can only measure deflection at discrete points, and require traffic disruption. Therefore, because of their advantages, in the recent years, non-destructive techniques are increasingly used by infrastructure managers to monitor pavements (e.g., speed of execution, accessibility, low interference with traffic). These monitoring systems allow both the traffic and environmental data to be collected without interrupting traffic flows, and, at the same time, they allow the pavement response to be continuously measured. They have aroused considerable interest from the scientific community, and substantial investments have been made in order to develop instrumented pavements with various embedded in situ sensors. Sensor measurements can also be used to determine the type of vehicle passing by, and the corresponding travel speed [33]. In the literature, several systems to monitor pavement conditions have been designed, based on built-in detection networks with an efficient combination of various sensors (e.g., horizontal and vertical strain gauges, load cells, geophones, accelerometers, thermocouples, humidity sensors, fiber optic sensors, noise detection sensors, and wireless data loggers for data storage). Using geophones and accelerometers, it is possible to calculate deflections by means of a single or double integration of the recorded measurements, respectively. Due to the non-realistic deflection values obtained with simple integration, [34,35] proposed an original signal processing procedure to obtain them, and then to assess the elastic modulus of pavement layers by means of back-calculation procedures.

In [36], horizontal strains are used to calculate the pavement moduli. Cafiso et al. [37] used a pre-polarized, omnidirectional, acoustically isolated microphone to collect the vibro-acoustic signatures of road pavements and correlate them to the elastic moduli obtained from FWD. The proposed solution could be used to continuously monitor the downgrading of asphalt road pavements under traffic. In [38], vibro-acoustic spectra are used to obtain information about the presence and severity of the cracks in the road pavement section. In [39,40,41], optical fiber technology is introduced, developed, and tested to measure the stress–strain state and temperatures inside asphalt pavements [42]. Also, airport concrete pavements have been analyzed to correlate distresses with both traffic- and temperature-induced loads with similar advanced technologies [43]. Most of the studies in the literature do not consider the residual service life of the network [44], whose lifespan depends on the lifespan of the sensor components. Self-healing and even more self-powered sensors [45] could significantly reduce the costs for the installation of sensors and pavement management. In order to aid the pavement management activities, several researchers [46,47,48] have focused on implementing sensors in road pavements with wireless data acquisition systems that allow continuous and remote monitoring.

In the airport field, some experiences with embedded sensors in runway pavements were carried out in the USA [49], and in Italy at Cagliari airport [50,51,52,53].

This paper aims to present the design criteria for a system that monitors the performance of an airport runway in real time. Its pavement is trafficked by military airplanes, and it is composed of concrete thresholds and an asphalt central runway. The proposed model permits the following:

- To analyze the structural response of the pavement;
- To prepare a pavement management protocol (i.e., PMS);
- To implement the management of the structural monitoring system by setting up a web monitoring system (WMS) with a dedicated app.

Particularly, the thermometers, strain gauges, pressure cells, and displacement transducers in the pavement aim to measure the temperature at different depths, the loads, and the deflections produced by the passage of aircrafts. The collected data will be transmitted, via the central unit, to a remote server, in order to save, review, and process them. An interpretative physical model of the pavement response to thermal and mechanical stresses will also be implemented. This will allow the pavement performance decay to be simulated, and will contribute to setting up the pavement management protocol. Finally, a special app will be developed to access the data in real time.

## 2. Materials and Methods

In order to design an innovative system to monitor airport pavements and define their pavement management system, a structural model must be adopted to describe stress–strain conditions of a pavement. For flexible pavements, multilayer linear elastic models from Boussinesq [54] and Burmister [55] theories are the most commonly used models to describe mechanical performances of pavement layers. However, it has to be noticed that the different pavement layers show behavior far from that of the elastic linear one. For this reason, a viscous elastic model or finite element models (FEM) are increasingly employed; they also take into account non-linear elastic performances of asphalt materials. For rigid pavements, the most common model used relies on a thin, infinite or semi-infinite plate resting on a bed of springs (Winkler foundation), and the resolution of this model is based on the Westergaard theory [56]. As for flexible pavements, even for the rigid ones, the FEM are emerging [57,58]. Particularly, FEM analyses allow thermal stress–strain conditions that are often overlooked by design guidelines to be taken into account (e.g., [59]). Therefore, software based on different mechanistic theories [60] are currently used to analyze mechanical performance of pavements (e.g., [61,62,63,64,65]).

### 2.1. Asphalt Pavement Verifications

The stresses and strains of the asphalt pavements were calculated according to the elastic multilayer theory and the pavements were verified with Miner’s law, considering the experimental relations described in the next paragraphs to calculate the allowable number of repetitions before fatigue rupture in bound layers and rutting in unbound layers.

#### 2.1.1. Fatigue Verification

For flexible pavements, Equation (1) describes a fatigue law [66].

(1) $$N_{f}=k\cdot {\left(\frac{1}{\varepsilon_{t}}\right)}^{n}\cdot {\left(\frac{1}{E}\right)}^{m}$$

where *N_f_* is the fatigue life; $\varepsilon_{t}$ is the maximum tensile strain induced by a traffic load; *E* is the elastic modulus of the layer; *k*, *n*, and m are constants depending on the material. In this study, Equation (2) will be adopted to analyze asphalt layers [67].

(2) $$N_{f}=0.00432\cdot k\cdot C\cdot {\left(\frac{1}{\varepsilon_{t}}\right)}^{3.9492}\cdot {\left(\frac{1}{\left|E^{*}\right|}\right)}^{1.281}$$

where the following apply:

- $k=\frac{1}{0.000398+\frac{0.003602}{1+e^{11.02-3.49h}}}$ for bottom-up cracks, where *h* is the thickness;
- $k=\frac{1}{0.0001+\frac{29.844}{1+e^{30.544-5.7357h}}}$ for top-down cracks;
- $C=10^{M}$;
- $M=4.84\cdot \left(\frac{V_{b}}{V_{v}+V_{b}}-0.69\right),$ where $V_{v}\;\mathrm{and}\;V_{b}\;$are the voids and bitumen volume, respectively.

*N_f_* is the fatigue life for the examined load. In this study, the fatigue verification complies with Miner’s law [54] according to Equation (3).

(3) $$\overset{P}{\underset{i=1}{\sum }}\overset{A}{\underset{j=1}{\sum }}\frac{n_{i,j\;}}{N_{i,j\;}}\leq 1$$

where $n_{i\;}$is the number of passages of the *i*-th axle; $N_{i\;}$is the number of repetitions of the *i*-th axle that causes fatigue; *A* and *P* are the total number of load configurations and period of analysis, respectively. If Equation (3) is satisfied, the pavement did not reach its fatigue threshold.

#### 2.1.2. Rutting Verification

Equation (4) describes rutting law [68], as follows:

(4) $$\frac{\varepsilon_{p}}{\varepsilon_{r}}\left(n\right)=\alpha \cdot n^{\beta }$$

where $\varepsilon_{p}$ is the vertical permanent deformation at the n-th cycle; $\varepsilon_{r}$ is the vertical elastic deformation induced by the load; α and β depend on material, temperature of asphalt layers, or moisture of granular layers. Moreover, according to [69], in this study the rutting curve for asphalt layers proposed by NCHRP 1-37A [70] has been adopted (Equation (5)).

(5) $$\varepsilon_{p}=k\cdot \varepsilon_{r}\cdot 10^{-3.4488}\cdot T^{1.5606}\cdot N^{0.479244}$$

where the following apply:

- $k=\left(C_{1}+C_{2}\cdot z\right)\cdot 0.328196^{z},$ where *z* is the generic depth;
- $C_{1}=$ −0.1039$\cdot h^{2}+2.4868\cdot h-17.342$, where *h* is the thickness;
- $C_{2}=$ 0.0172$\cdot h^{2}-1.7331\cdot h+27.428$.

The maximum rutting depth assumed in this study is 25 mm [14]; higher values require maintenance work.

#### 2.1.3. Thermal Rupture Verification

Thermal cracking is caused by rapid decrease in air temperature; when tensile stresses induced by thermal gradient are greater than tensile strength of asphalt they cause the rupture. Starting from the maximum temperature of the day, to which a zero strain value corresponds, at each constant interval ∆*T*, the induced tensile stress is determined according to Equation (6) [71].

(6) $$∆\sigma \left(t,∆T\right)=\alpha ∆TS_{m}\left(t,∆T\right)$$

where α is the coefficient of the linear thermal expansion of asphalt and $S_{m}$ is the asphalt stiffness at (t,∆T). Therefore, the overall increase in stress level is calculated according to Equation (7).

(7) $$\sigma \left(t,T\right)=\alpha \sum \{∆T\cdot S_{m}\left(t,∆T\right)\}$$

The verification consists of the comparison between σ(t,T) and the tensile strength $\sigma_{t}\left(T\right)$ of the material at its minimum daily temperature.

### 2.2. Concrete Pavement Verifications

The stresses and strains in the concrete pavements were calculated by an FEM software; the maximum tensile stresses are localized at the bottom of the slab both for a load at the interior and at the edge of the slab or at the top of the slab for a load at the corner. It has to be noticed that thermal conditions in concrete slabs also affect strains and stresses [72,73], so ignoring them exposes the concrete pavements to unacceptable risk of premature failure [74]; stresses imposed by temperature must be considered as important as stresses inducted by traffic.

#### Fatigue Verification

As conducted for the flexible pavements, the concrete ones were also verified with Miner’s law, considering the relations described below to calculate the allowable number of repetitions before fatigue rupture in the concrete slab.

Equation (8) describes fatigue law for concrete slabs, as follows:

(8) $$\log\left(N_{f}\right)=\alpha +\beta {\left(\frac{\sigma }{M_{R}}\right)}^{\delta }$$

where σ is the maximum tensile strain induced by load and temperature; $M_{R}$ is the concrete modulus of rupture; α, β and δ are regression coefficients that depend on the adopted experimental curve. Equation (8) is valid for tensile stresses that are no less than 0.5 Rctf (i.e., the flexural traction resistance) because under this tensional value fatigue does not occur. In this study, Equation (9) [75] and Equation (3) have been adopted.


(9) $$\log N_{f}=2\cdot {\left(\frac{M_{R}}{\sigma }\right)}^{1.22}+0.4371$$

### 2.3. Case Study Sensors

In this study, sensors to measure temperature, load and deflection (i.e., thermometers, strain gauges, and pressure cells) will be installed in a runway pavement. The adopted selection criteria focused on the following:

- Very high sampling frequencies with dynamic acquisition of measurements (i.e., deformations, pressures and temperatures);
- Methods of sensors’ installation depending on the pavement type and its temperature during its service life from construction phase;
- Order of magnitude of the expected measurements;
- Reliability of collected data;
- Durability of the instruments.

#### 2.3.1. Thermometers

**Figure 1:**
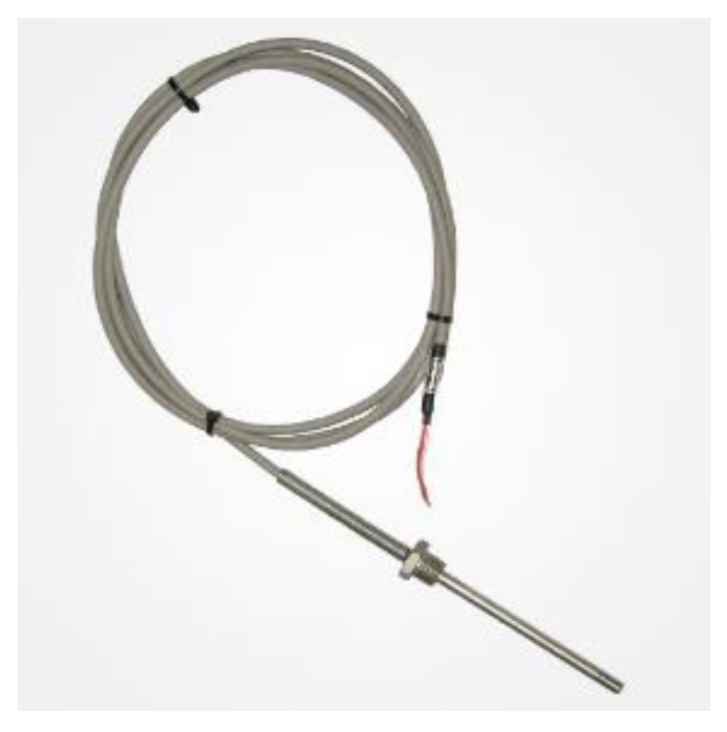
Thermometer.

To assess the influence of thermal effects on the measurements and on the examined pavement, temperature is a very important parameter to monitor. The adopted thermometers (Figure 1) are sensors with platinum RTD (resistance thermal detector) thermistor PT100 and are housed in a shock-resistant stainless-steel body to be embedded in asphalt and concrete. The instruments are stainless-steel cylinders, inside which the sensor is embedded and sealed with resins that transmit heat well. The operating temperature ranges from −75 °C to +250 °C. Temperature string probe is available for multiple thermal measurements in boreholes, trenches or to be embedded.

The temperature string probe has an output with a 50 m cable in PFA, and an ODU connector to link it to the acquisition data system.

#### 2.3.2. Strain Gauges

The property called “deformation” is considered to be the ratio of the change in length to the original, not stressed length of an object. Strain gauge sensors can measure this length change caused by an external force and convert it into an electrical signal, which can then be analyzed. This occurs because a strain gauge sensor experiences a change in resistance proportional to elongation or compression and it can measure the amount of strain inside a pavement layer under dynamic loading (i.e., passing vehicles). According to the pavement type, these sensors are composed of aluminum or inox steel, when embedded in asphalt or concrete, respectively. The sensitive part is situated in the central bar and contains 4 active strain gauges connected in a full-bridge configuration; each edge of the central bar is connected to a metal strip.

**Figure 2:**
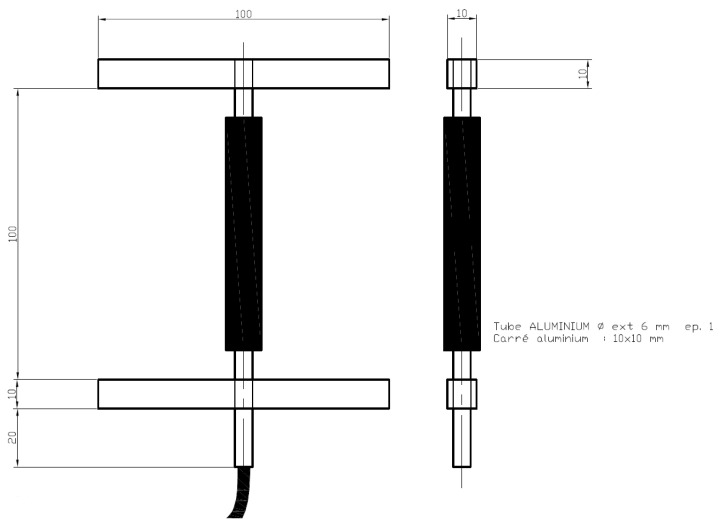
Asphalt strain gauges.

For asphalt layers, they are as shown in Figure 2.

They are suitable for heat bonding and have an IP67 degree of protection. The maximum operating temperature is 180 °C.

**Figure 3:**
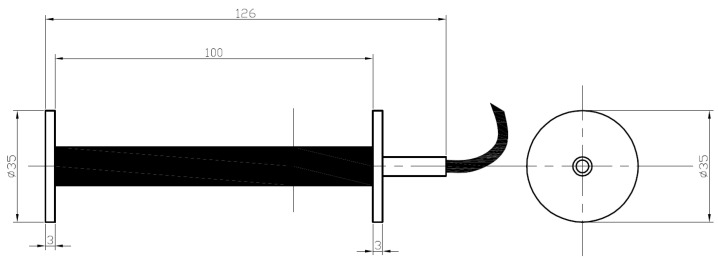
Concrete strain gauges.

For concrete layers, they are as shown Figure 3.

They are suitable for heat bonding and have an IP68 degree of protection, suited to an alkaline environment. The maximum operating temperature is 100 °C.

According to Equation (10), the gauge factor (GF) refers to the variation in resistance (Δ*R*) caused by the deformation on the native resistance (*R*) of the sensor, divided by the deformation itself (ε), as follows:

(10) $$\mathrm{GF}\;=\;(\frac{∆R}{R})/\varepsilon$$

#### 2.3.3. Pressure Cells

**Figure 4:**
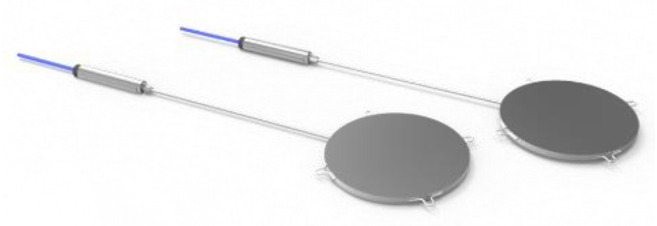
Pressure cells.

Pressure cells are used to measure compressive stress in the pavement, particularly in the unbound layers. They will be placed at the interface between the different pavement layers. The sensors are shown in Figure 4.

For asphalt pavements, pressure cells have a diameter of 127 mm and their operating principle is diaphragm-based. The diaphragm cells consist of a rigid ring that supports a diaphragm. The application of a load causes a deflection of the diaphragm, which is measured by a strain gauge; the correlation between diaphragm deflection and compressive stress depends on the adopted instrument and it is given by calibration curves adopted by the producer. The sensors have a full scale of 6 MPa and a maximum operating temperature of 180 °C. For concrete pavements, the sensors are made of two inox steel circular plates, welded together around their perimeter. The annular space between these plates is filled under vacuum by deaired oil. The pressure pad is connected by means of a stainless-steel tube to the transducer, forming a closed hydraulic system. This has a diameter of 230 mm and a full scale of 6 MPa, and a maximum operating temperature of 100 °C.

## 3. Case Study

A remote system has been designed to monitor a flexible and rigid runway of an Italian military airport, herein not disclosed, due to reasons of confidentiality. The central part of the runway pavement is composed of 10-cm-thick surface course (4 cm wearing course and 6 cm binder course), 15 cm asphalt base, 30 cm cement-treated subbase, and 35-cm-thick granular subbase (Table 1). Further, the thresholds have 29-cm-thick concrete slabs, laid on 20-cm-thick cement-treated subbase and 40-cm-thick granular subbase (Table 2).

The traffic is mainly composed of C130J-Hercules, considering the following characteristics: a maximum take-off weight equal to 79.38 t, tire pressure of 0.74 MPa, and 1500 yearly movements. The subgrade CBR is equal to 6.5%.

**Figure 5:**
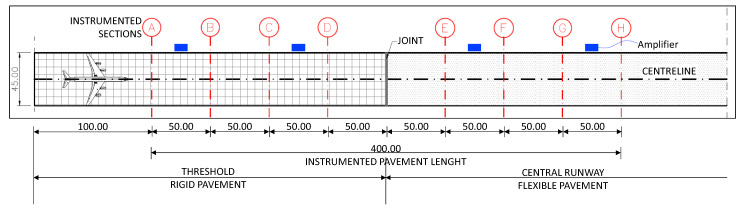
Layout of the runway instrumented sections.

In order to define the correct transversal and longitudinal positions of the chosen sensors, reference is made to the design plane C130J [76], a typical vehicle for the air fleet in the military airport. The sensors will be installed to monitor the most frequently damaged areas of the pavement (i.e., threshold and first sections of the runway). In the examined airport, the thresholds are paved with concrete slabs, while the runway is paved with asphalt pavement. Eight transversal sections, spaced 50 m apart, will be instrumented; there will be four in the rigid pavement and four in the flexible one (Figure 5).

**Figure 6:**
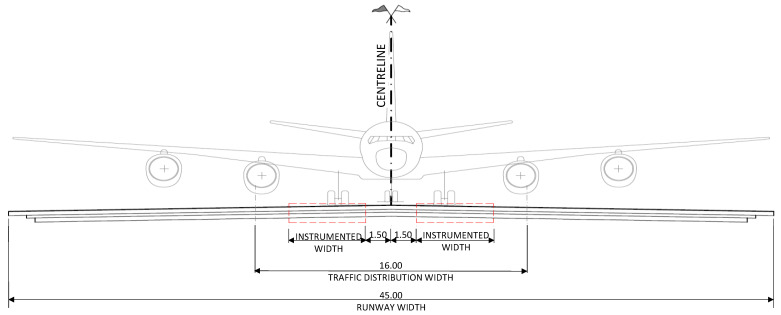
Transversal layout of sensors.

In each transversal section’s sensing technologies will be embedded, taking into account the dispersion of aircraft trajectories; the most heavily loaded section, of about 16 m wide and symmetrically distributed with respect to the runway centreline, has to be considered [77] (Figure 6).

**Figure 7:**
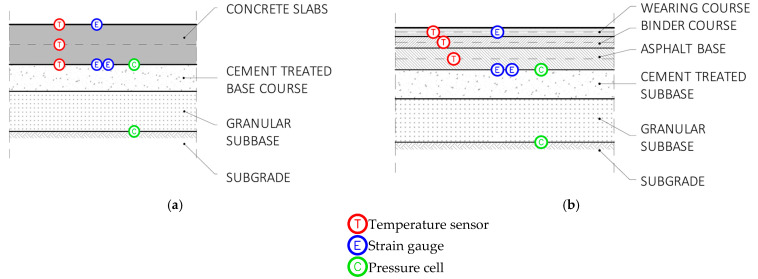
Sensors number and layout: rigid pavement (**a**); flexible pavement (**b**).

Figure 7a,b shows the embedded sensors in the rigid and flexible sections, respectively. With regard to Figure 5, sections A to D refer to the rigid pavement and sections E to H to the flexible one.

The acquisition system allows the pavement conditions to be continuously monitored, and, through a trigger function, it is able to record them, only during the passage of the aircraft. In this way, the collected data will be correlated to the thermal and mechanical loads on the runway. However, the sensors need an initial calibration, then the physical quantity required is read directly; the engineering evaluation of the instrument is a direct comparison between the recorded measurement and that of the threshold deriving from a calculation model (FEM). If anomalies are detected, an attempt is made to characterize the defect and the damage that may have occurred. To take into account the effect of temperature and relative moisture, the inelastic component of deformation shall be purified from the readings.

### 3.1. Data Acquisition System

It is planned to use a particular data acquisition system, composed of amplifiers, connection accessories, switches for cable connection and measurement synchronization, and a software for data acquisition, visualization, analysis, and reporting measurement data. The amplifiers have the purpose of transforming the different output signals, derived from the various types of sensors used in this study, into higher-level amplified electrical signals. The software is useful to view, analyze, and store data during the measurement. It allows GPS data to be viewed on maps, data to be exported and stored in files in standard formats, the comparison of data sets, computation, and the analysis of signals.

### 3.2. Monitoring System Management

#### 3.2.1. Data Processing

The objective of the monitoring system is to control the conditions of the structure, which, in this case, is the airport runway pavement, with respect to both the serviceability and ultimate limit states; among these, the failure of the operating conditions is of great interest for the infrastructure manager. The system is designed to keep the value of some significant parameters under control, in particular, the following: local deformations of the pavement under the wheel load and inelastic ones due to thermal effects; the pressure related to the passage of the aircraft, and, finally, the temperature. The sensors have been identified to be efficient in the cases of large variations in deformation (cracking), high pressures, and bad environmental conditions (i.e., high temperatures of asphalt during the pavement building phase). The measurements recorded by the system will be pretreated to improve the quality of the data through, for example, the elimination of the effects of noise and possible “spikes”, transformation into engineering values, validation of the data, also exploiting correlations with other measurements, and thermal compensation.

#### 3.2.2. Interpretative Models

The obtained data will be interpreted through numerical models and probabilistic models. The numerical models realized with finite element software allow a correlation between the experimental measurement and the theoretical response. The FEM must be previously calibrated in order to accurately reproduce the real behavior of the pavement. For this purpose, it is necessary that the measurements are processed for a period in which the pavement can be considered intact. Once the theoretical reference model has been defined, any differences that subsequently appear between the response of the real structure and the model, exceeding the tolerances defined during calibration, can be interpreted as anomalies. In cases of anomalies, an automated procedure will be defined that aims to update the model according to the measures recorded, in order to identify and estimate the phenomenon of degradation in situ. In addition, damage identification will also be assessed through a Bayesian probabilistic approach, in which several indicators contribute to define the probability that the pavement has a state of damage. The different indicators result from the analysis of the recorded data, and from the application of a process that provides different strategies that, together, reduce the possibility of producing false alarms, positive or negative (i.e., to report damage that is not present, or not to report damage, even in the case of actual damage).

#### 3.2.3. Alert Thresholds and Management Protocol

**Figure 8:**
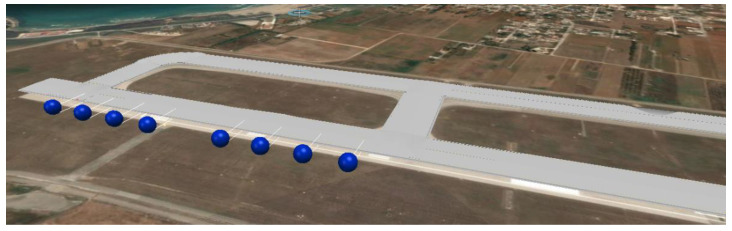
iTwin model of the examined airport.

Downstream of the initial training period, and after calibrating the finite element model, alert thresholds will be established on the basis of the probability of exceeding limit states, in correspondence of which countermeasures will be defined and planned. The collected data will be managed through the Bentley Infrastructure Digital Twins (iTwin) technology that allows the sensors’ data along the runway to be monitored, helping to reduce the associated risk and the liability resulting from asset failures. This service permits digital, up-to-date, virtual representations of assets to be created and managed, to view the project 24/7, from anywhere, in order to know how it performs. Figure 8 shows the iTwin model for the under-study airport.

**Figure 9:**
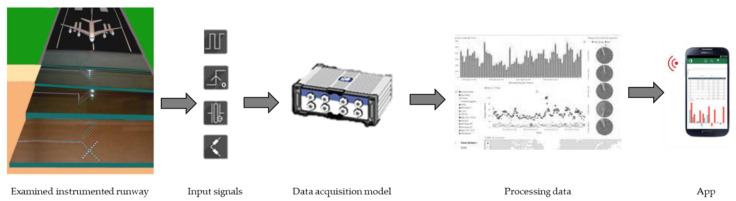
Operating scheme of the proposed methodology.

The data processing and model updating software, within which the alert thresholds will be managed, will be linked to a management protocol. This is defined in agreement with the airport manager, and contains a list of operations to be carried out remotely and in situ to verify the functionality and safety of the pavement; it also contains a list of countermeasures to be implemented if the alert thresholds are exceeded. All the data will be available in real time on an app, protected and dedicated to the manager, which will contain the information material related to the monitored pavement (images, descriptions, graphics, operating manual, and emergency management manual) and the database of data updated at regular intervals, as shown in Figure 9. The database will be consulted in interactive mode, and the user will be able to create customizable graphs with the response quantities of interest and verify the surplus in the thresholds corresponding to the different limit states previously defined. Notifications will indicate the occurrence of anomalies and the countermeasures to be implemented.

## 4. Discussion

A fully functional airport surface is an essential condition for guaranteeing adequate safety and comfort conditions during its entire service life. Therefore, the achievement and maintenance of some fundamental requirements regarding the structural performance of the pavement, such as the possibility to transmit aircraft loads to the subgrade and resistance to structural degradation, are of primary importance. The operator must implement an APMS to properly manage the pavements, monitoring their performance over time.

Traditionally, discontinuous and manual monitoring systems have been adopted so far. This paper presents an innovative remote sensing system to continuously monitor airport pavements, without interfering with the circulation. It is composed of hardware and software components that collect data on temperature, strain and stress at different depths under the pavement surface. With this purpose, thermometers, pressure cells, and strain gauges will be embedded in the pavement layers; their characteristics depend on the material to be monitored. The sensors will be installed in the most frequently damaged areas of the pavement (i.e., threshold and first sections of the runway). In the examined airport in Italy, the runway thresholds are paved with concrete slabs, while the central runway is paved with asphalt pavement. The data recorded by the proposed system will be processed using proper software, in order to implement an app that is able to inform the pavement manager in real time. In this way, a system that can provide useful information for the correct processing of an APMS can be developed. The actual pavement conditions, and their evolution over time, will be collected in an object/relational database that allows a decision support system related to technically and economically sustainable management strategies.

The proposed remote monitoring system could be implemented in different airport pavements and could be adapted to different transport infrastructures, in order to monitor their performances over time. Particularly, the technical properties of hardware should be deeply investigated to identify the best option for varying construction and service conditions. Further research could investigate the durability and maintenance procedures of sensors, according to the available technologies.

## 5. Conclusions

Airport pavements play a pivotal role in the management of safe and regular movements; functional and structural characteristics are to be investigated, according to scheduled monitoring activities. In the recent years, non-destructive testing replaced traditional and destructive testing, especially for road pavements. On the other hand, in the airport sector, this approach is still used a little. 

This paper presents a technical proposal for monitoring the thermal and mechanical stresses of a military Italian runway pavement, whose design aircraft is C130 J-Hercules. The runway to be instrumented is composed of thresholds paved with jointed plain concrete slabs, while the runway is paved with asphalt pavement. Overall, eight sections—four of them for each pavement type—will be instrumented with the following embedded sensors: thermometers, strain gages, and pressure cells. The data coming from the different sensors will be collected, stored, and then treated to be available in an easy-to-read and easy-to-operate remote system. Fatigue, rutting, and thermal rupture verifications will be calculated according to the analytical theories available in the literature. The results from the implementation of the decay curves will populate a dedicated app, through which the airport manager could have access to real-time pavement conditions and could be alerted of exceeding threshold values. Particularly, the proposed methodology allows the pavement manager to obtain decision support throughout the design, construction, and operations, and reduce the cost and time of delivering projects and managing assets. 

## Figures and Tables

**Table 1:** Mechanical performances of materials in asphalt pavements.

Material	Parameter	Value	Unit
asphalt–wearing course	stiffness modulus, 25 °C	3600	MPa
	indirect tensile strength	1.92	MPa
asphalt–binder course	stiffness modulus, 25 °C	3000	MPa
	indirect tensile strength	1.84	MPa
asphalt–base course	stiffness modulus, 25 °C	2000	MPa
	indirect tensile strength	1.75	MPa
cement treated mix	cylindrical characteristic compressive strength, 7 days	3–7	MPa
	indirect tensile resistance, 7 days	0.45–0.85	MPa
granular mix	liquid limit	<25	%
	plasticity index	0	%

**Table 2:** Mechanical performances of materials in rigid pavements.

Material	Parameter	Value	Unit
concrete	cube characteristic compressive strength, 28 days	≥45	MPa
	modulus of rupture, 28 days	≥4.1	MPa
cement treated mix	cylindrical characteristic compressive strength, 7 days	3–7	MPa
	indirect tensile resistance, 7 days	0.45–0.85	MPa
granular mix	liquid limit	<25	%
	plasticity index	0	%

## Data Availability

The data presented in this study are available on request from the corresponding author. The data are not publicly available due to confidentiality reasons.
